# Developing an Evidence-Based Epilepsy Risk Assessment eHealth Solution: From Concept to Market

**DOI:** 10.2196/resprot.5389

**Published:** 2016-06-07

**Authors:** Craig Newman, Rohit Shankar, Jane Hanna, Brendan McLean, Alex Osland, Cathryn Milligan, Abbie Ball, Caryn Jory, Matthew Walker

**Affiliations:** ^1^ Neuro-Cognitive Research Group (NeuroCoRe) Plymouth University Peninsular School of Medicine and Dentistry (PUPSMD) Plymouth United Kingdom; ^2^ Exeter Medical School Exeter United Kingdom; ^3^ SUDEP Action Oxfordshire United Kingdom; ^4^ Royal Cornwall Hospital Trust Truro United Kingdom; ^5^ Neuro-Cogntivie Research Group (NeuroCoRe) Plymouth University Peninsular School of Medicine and Dentistry (PUPSMD) Plymouth United Kingdom; ^6^ Cornwall Partnership NHS Foundation Trust Bodmin United Kingdom; ^7^ UCL Institute of Neurology London United Kingdom

**Keywords:** eHealth, mhealth, mobile app, epilepsy, SUDEP, self-management, self-monitoring, smartphone

## Abstract

**Introduction:**

Sudden unexpected death in epilepsy (SUDEP) is possibly the most common cause of death as a result of complications from epilepsy. The need to educate and regularly review risk for all patients with epilepsy is paramount, but rarely delivered in actual clinical practice. Evidence suggests that education around SUDEP and modifiable risk variables translate into better self-management of epilepsy.

**Objective:**

We aimed to develop and implement an eHealth solution to support education and self-management of risks, in epilepsy.

**Methods:**

We undertook an innovation pathways approach, including problem identification, feasibility assessment, design, implementation, and marketing. People with epilepsy were provided a smartphone-based app (Epilepsy Self-Monitor, EpSMon), which translates the clinical risk assessment tool into an educational and self-monitoring platform, for the self-management of epilepsy.

**Results:**

Results include the success of the marketing campaign, and in what areas, with an estimated reach of approximately 38 million people. EpSMon has proved a success in academic and clinical circles, attracting awards and nominations for awards. The number of users of EpSMon, after 3 months, turned out to be lower than expected (N=221). A 4-month trial of the app in use in the United Kingdom, and the success of the marketing strategy, point to necessary changes to the model of delivery and marketing, summarized in this paper. These include the marketing message, user cost model, and need for the availability of an Android version.

**Conclusions:**

EpSMon has proven a success in respect to its reception by academics, clinicians, stakeholder groups, and the patients who use it. There is work needed to promote the model and increase its acceptability/attractiveness, including broadening the marketing message, increasing its availability, and reducing its cost. Future development and promotion of the tool will hopefully inform iterative design of its core features for a receptive audience and lead to increased uptake as it is launched worldwide in 2016.

## Introduction

### Background

Epilepsy is one of the most common neurological disorders globally affecting 5 to 40 people per 1000 population [1]. Epilepsy affects approximately 50 million people throughout the world. It has been estimated that 10% of the burden of brain and mental disorders in the world is caused by epilepsy. This calculation includes premature deaths and the loss of healthy life due to disability [2].

Sudden unexpected death in epilepsy (SUDEP) is the most important direct epilepsy-related cause of death [3]. SUDEP is possibly the most common cause of death as a result of complications from epilepsy, accounting for between 7.5% and 17% of all epilepsy-related deaths and [4] 50% of all deaths in refractory epilepsy [5]. Sudden death is 20 times higher in people with epilepsy (PWE) than the general population. Epilepsy is the 5th highest cause of life-years lost and the public health burden of SUDEP alone is estimated as second only to stroke among neurological conditions [6]. Forty-two percent of all deaths are considered avoidable [7]. Consequently, the National Institute for Health and Care Excellence epilepsy guidelines 2004 and 2012 [8] recommend discussion of SUDEP with newly diagnosed PWE. This is rarely delivered and until recently only 4% of PWE had a recorded SUDEP discussion [9].

There is robust evidence to suggest that knowledge relating to modifiable risk factors would help empower the patient to take responsibility toward his well-being in managing his condition [10-12]. Recent studies have shown that factors influencing SUDEP and other direct causes of epilepsy death overlap [13-15].

### Aim

Development of a patient-centered eHealth solution to reduce risk of SUDEP and educate PWE around risk. Utilization of quality improvement and iterative cycles of development to evidence the proposed solution.

## Methods

### App Conceptualization

In the conceptualization stage of a patient-administered eHealth alternative to the consultant-administered checklist, it was important to identify how this might fit alongside existing care pathways and where, if anywhere, a clear gap in service delivery was observed. Consultation was undertaken with a specialist national Epilepsy charity (SUDEP Action), a specialist general practitioner (GP) commissioning group in Cornwall, UK and both national academic and consultant specialists in epilepsy.

This steering group identified the removal of the primary care–based Quality Outcomes Framework financial support for annual epilepsy reviews, provided by GPs, would likely translate into a significant reduction in epilepsy reviews in the community for patients not identified as at risk. A service pathway was drafted, which included an eHealth self-monitoring option, for patients to self-administer, which could act as a triaging tool alongside existing primary care models.

### Stepped-Care Model Approach

A rapid informal review of eHealth solutions delivered in other sectors revealed a range of options for support, including general education, risk-specific educational interventions, prompts to seek help, and automated triggering of community or secondary care service support. Against the current level of support for PWE with ongoing seizures, where it is currently their own responsibility to seek help when they perceive a need, the steering group perceived that support in the ability to identify when this need is present was most relevant. This is reinforced by earlier reported findings [16] in which coroner data for SUDEP-related deaths identified that only 20% of patients had sought medical contact with an epilepsy specialist in the period of 12 months prior to their deaths. The evidence indicated a 3-month high-risk period for people whose risk profile deteriorates, as identified by the checklist, and so the identified eHealth need was the provision of an informative risk assessment at prompted 3-monthly intervals.

### Technology Identification

The steering group assessed the potential risks and benefits of differing technologies to support this eHealth solution, including a systematic review of existing seizure detection methods [17]. Primarily, the goals of the project required ease of use, accessibility, effortless international dissemination, notification capabilities, and data collection capabilities (to support the ongoing development of the checklist). A mobile app was selected due to the ongoing surge of take-up of these devices (6.9 billion mobile phone prescriptions worldwide with a forecast of 5.6 billion smartphone subscriptions by 2019, Global mobile statistics report - 2014 [18]), the tendency for owners to carry their device at all times, the numerous notification options and the expansive range of data collection options including take-up and user retention data.

### App Design (EpSMon – Epilepsy Self Monitor)

**Figure 1:**
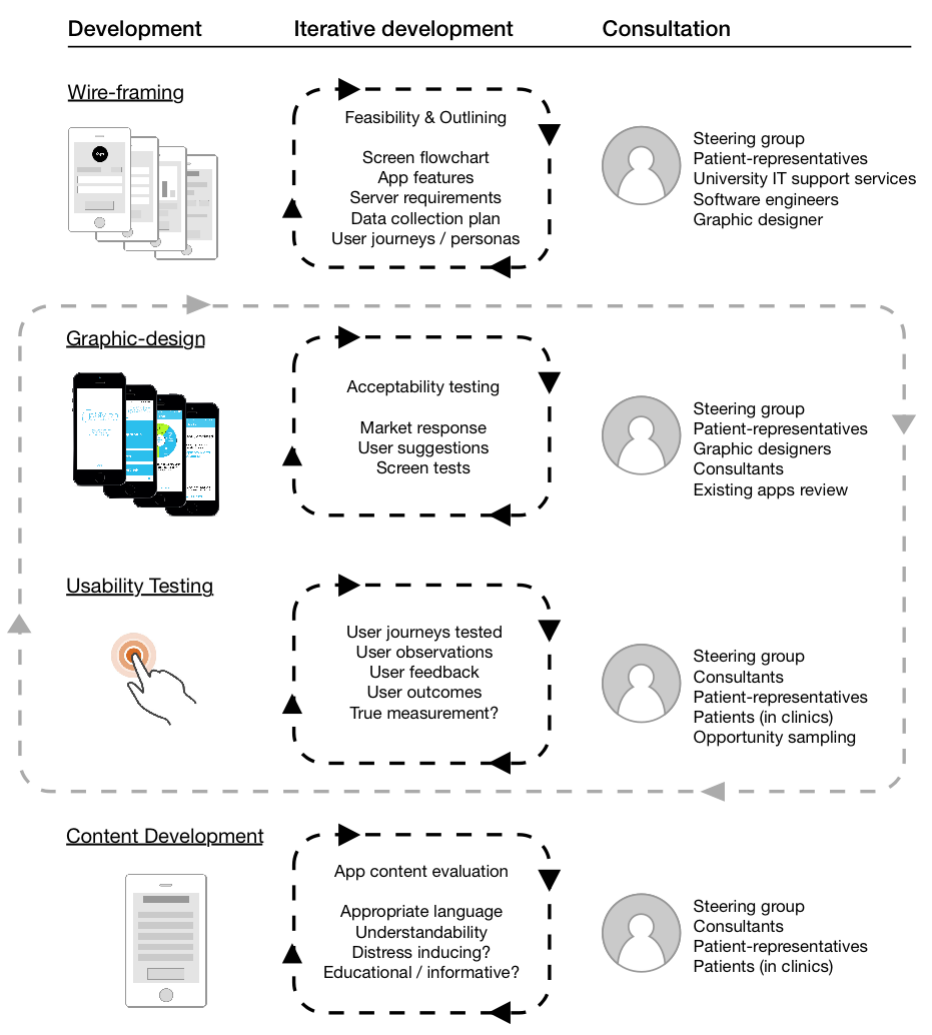
Cycles of iterative development undertaken in creating EpSMon.

The development of the App content was iterative, cycling on the basis of specialist input across a range of development themes: the consultant steering group, a patient-representative group (recruited by the charity), University information technology (IT) support services and patients of volunteer clinicians were repeatedly involved in providing feedback across a range of development stages, (summarized in Figure 1)

A wire-frame (flowchart of screens, features, and server activity) was initially developed by a local University-based team who specialize in the development of apps for neurology and clinical services. Following this, a graphical representation mock-up was produced providing, first versions of the graphics in situ in a functioning App and the final Beta version prerelease (a Beta version being the version that is tested for coding bugs, all other features assumed complete). The target users for this tool span all ages and demographics and so the development team strived to access a diverse feedback group.

Usability testing (public and patient involvement, PPI): explored and observed how users experienced and used the app naturally and across a series of identified tasks. This research was led independently by the charity (to be published separately), software team, and specialist clinicians in clinics with patients. Feedback was reviewed by the steering committee with design modifications to the app when deemed appropriate.

In order to generate the content of the app, the translation of the checklist into a self-administered tool, supported by educational material, was led by the charity in liaison with a specialist patient consultation group and specialist clinicians and academics in this field. This content was reviewed in the first version of the app, by volunteer patient representatives, with edit recommendations made when deemed appropriate by the steering committee. The steering committee decided upon a minimum governance standard for the project, requiring that the checklist be taken through an annual update, requiring ongoing reviews of the literature. An update cycle was undertaken during the build of the app and the app content was updated accordingly.

Once version 1 of the app was built, beta testing was completed for bugs in its functioning by all supporting partners, and a range of patient representatives who had registered to support this activity on a prerelease invitation hosted on the charity’s website. Prior to release of the app to iTunes, the app’s code, user policies, terms of use, data protection protocols, and security protocols (data encryption, secure transfer, etc) were all reviewed by an IT specialist and legal services provided by the University partner.

Given the heterogeneous characteristics of the potential user population, being any individual with epilepsy and so any age, sex, or race – speculations about user preference or accessibility to devices seemed uninformative beyond national update statistics. Rather, it was decided to aim to release the app to the most popular platforms (Apple and Android) with the aspiration to expand to provide to Windows phones if resources allow.

### Key Feature Selection

Following the iterative design process, the app was completed with a range of features that are supportive to patients, service delivery and future research. The features included represent the minimum required to rapidly meet identified user needs and research model of the project, with additional features to be considered in future updates.

It was considered valuable to develop capability to support users in self-triaging their need to seek clinical contact, following an assessment of risk. The translation of the checklist into a self-assessment provides a robust evidence-based self-triaging tool that identifies risk change across regular time intervals. Rather than providing a binary health care approach, a stepped-care approach was designed into the app. EpSMon initially educates PWE around variables related to risk through regular exposure to the checklist. Secondly, succinct educational content is provided to support further queries around identified risk with the app designed to encourage access to this information. Finally, the app encourages contact with a GP when appropriate and queries whether this was adhered to at the next assessment. As an additional safety precaution, a telephone number is provided for people who are distressed or confused, direct to SUDEP Action.

Interventions in other disease areas frequently use a stratified approach, cutting off access to services at a particular percentage of risk of serious disease or death. The Checklist and EpSMon do not do this. The app replicates the checklist, in that it is designed to not include a cumulative score or percentage of risk so there is no cut off of risk, hopefully maximizing doctor and patient empowerment and risk-management. To maintain a quality risk-management and clinical governance approach, the app is designed to support research by consenting users into research and collecting data relating to user demographics, comorbidity, medication use (epilepsy, depression, and psychosis), risk profiles over time, and app usage.

### App Governance

After reviewing the government’s criteria for app medical device registration [19], it was considered that EpSMon does not meet entry requirements. EpSMon does not diagnose, monitor, prevent, treat, or alleviate a disease. Although the tool is called a monitor, this is a reference to the tool supporting the self-report of user’s perceptions about their current state that is used to prompt education or conversation with a clinician, no raw data is interpolated for clinical use. The app is not an alternative to standard treatment and nor does it interfere with or recommend any treatment plan, which is a stipulation (not met) for medical device status required by the Medicines and Healthcare products Regulatory Agency. It informs its users that the results are not to be considered as anything but suggestive. This position could change if the underlying questionnaire responses are developed into statistically weighted factors and the report process becomes more interpretative. This may also need further consideration when EpSMon is released to other countries, such as the United States.

While the safety checklist was developed for SUDEP, risk factors such as nonadherence and depression are relevant to epilepsy mortality generally. They bring a set of questions to the fingertips of doctors and patients that were developed from research on SUDEP. Many of the questions overlap with epilepsy mortality generally and the 2015 version of the Checklist agreed by a UK development group of experts includes the latest research on epilepsy fatality. These include questions on wellbeing as well as seizures.  The Checklist will be reviewed annually to enable updating with evidence on fatality. A question on comorbidity has already been identified as an area for recommended inclusion in 2017.  Country versions outside the United Kingdom will need additional information and country contextualization if there are no national guidelines in place.

## Results

### Launching the Epilepsy Self-Monitor App

EpSMon was launched to iPhone (UK only) in July 2015 at a cost of £1.49 for life-time access. A strategic marketing communications campaign supported the launch of the app, with public relations support from all partner organizations. Coverage achievements included 5× regional television news reports (audience estimates unavailable), 7× regional radio reports (estimated 1.5 million audience), 27× national and regional newspaper articles (estimated 33.2 million audience), 625 Facebook likes, 424 shares, and 255 clicks to the website (from a potential Facebook reach of 360, 000) and 110,300 ‘EpSMon’ retweets and 14,900 mentions. In total, the marketing communications campaign attained an overall reach of approximately 38 million people.

A full-time support phone line was indicated in the app, provided by SUDEP Action. Very little activity has been generated, with contact mostly relating to enquiries for an Android version of the tool.

Releasing EpSMon has been a very positive experience with unanimous support from clinicians, academics, and patients who have had contact. It has received awards at the International League Against Epilepsy conference, mass media interest, and two nominations to the Health Service Journal Innovation awards (HSJ) despite its infancy in respect to project duration. The almost nonexistent need for support, by users, encourages further expansion of the user base.

Since the launch, 221 users have downloaded the tool and registered, with 218 having assessed their risk. In addition, EpSMon has also been adopted into the National Epilepsy Commissioning Toolkit, alongside its parent checklist for clinicians. The app collects, with consent, users’ age, sex, epilepsy diagnosis duration, seizure type, medications use, questionnaire responses, and app-use frequency data for all users. This data will be used to both better understand the existing user base, for purposes of further project delivery, and to support the research that will further develop the risk checklist’s ongoing development.

### APP Modifications

The current user base, while modest, represents a test base for the project and has supported the identification of areas in which further work is needed. The first of these is cost. The introduction of a £1.49 fee was driven by a desire to test whether this would increase trust in the product, rather than a need to fund the project. Data indicates that 80% of visitors to the app download page do not purchase the app, which may be a consequence of this fee structure. The steering committee has chosen to remove this fee and to provide the app at no cost. This may, in addition, encourage GPs and other organizations to promote the tool without fear that there is a profit motive for the partners involved.

There is an imminent need to provide an Android version of the tool, based on feedback to the charity and the fact that 79% of smartphone ownership is Android [20]. This could translate into an immediate rapid uptake of EpSMon, especially in the context of this being provided at no cost.

The marketing campaign for EpSMon was built on earlier epilepsy work relating to safety, ‘Safety in your pocket.’  Feedback since the launch suggests that we may need to consider a multipronged approach.  Marketing strategies are currently being explored, with potential but resistant users. A second marketing communications campaign is planned for mid 2016, with news of the app now being free and available for Android and new Apple users. 

Discussions are underway with potential US supporters who can provide marketing support into a US market in early 2016. This will involve the bundling of a Spanish translation of the tool. A translation proforma has been developed, which will be provided to a translation agency in the near future.

## Discussion

### Principal Findings

The development of EpSMon and its first phase of implementation has been successful. In only 4 months the tool has been adopted into the commissioning toolkit for the National Health Service and has been nominated as a finalist in two categories of the UK’s prestigious HSJ. The project has also received recent prizes at the International League Against Epilepsy for best poster and best presentation. The reception from academics, clinicians, families, and users is to date unanimously positive and supportive.

The take up of the app has been modest, although this likely reflects a cocktail of challenges that will require a responsive and reflective approach, beyond the need for an Android version summarized above. The literature relating to SUDEP in many ways characterizes both community and clinical level denial or ignorance as to the need to monitor risk of death in PWE. Marketing to a potential user base who are unaware of, or disconnect from, the narrative of risk in epilepsy is a challenge. The EpSMon project will seek to engage with clinicians, who already appear very receptive, and user groups to develop marketing strategies that engage people in the community, GPs, and specialist clinicians. In addition, work is underway to embed EpSMon into standard advice practice of GPs, through research trials to demonstrate the efficacy of this approach and specialist GP e-training (expected implementation in spring 2016).

### Conclusions

Although this project appears U-centric, SUDEP is a global phenomenon. There is a higher prevalence of epilepsy in economically poorer and developing countries than economically developed countries [21]. In such poor countries, priority of safety and ignorance is rife. Presence of a cost-effective solution such as the app could save lives. The initiative toward making this app free to use, and housed within a research collaborative model, will hopefully invite opportunities to create impact in these contexts, through further development and uptake of EpSMon. Further usability research, academic publication of the tool’s merits, and developments alongside a strong research strategy will hopefully further support the success of this project into the future and beyond the United Kingdom.

## Abbreviations

EpSMon: epilepsy self-monitor
GP: general practitioner
HSJ: Health Service Journal Innovation awards
IT: information technology
PPI: public and patient involvement
PWE: people with epilepsy
SUDEP: sudden unexpected death in epilepsy
